# Impact of pneumococcal conjugate vaccine on invasive pneumococcal disease in children under 5 years of age in the Czech Republic

**DOI:** 10.1371/journal.pone.0247862

**Published:** 2021-02-26

**Authors:** Jana Kozakova, Pavla Krizova, Marek Maly

**Affiliations:** 1 Department of Air-Borne Bacterial Infections, National Reference Laboratory for Streptococcal Infections, Centre for Epidemiology and Microbiology, National Institute of Public Health, Prague, Czech Republic; 2 Department of Air-Borne Bacterial Infections, Centre for Epidemiology and Microbiology, National Institute of Public Health, Prague, Czech Republic; 3 Unit for Biostatistics, National Institute of Public Health, Prague, Czech Republic; Universidade de Lisboa Faculdade de Medicina, PORTUGAL

## Abstract

**Introduction:**

The aim of this study is to analyse the impact of vaccination of infants with pneumococcal conjugate vaccine (PCV) on the incidence of invasive pneumococcal disease (IPD) in children under 5 years of age in the Czech Republic.

**Material and methods:**

The present study includes all IPD cases reported in children aged 0–4 years within the surveillance program in 2007–2017. The impact of PCV is analysed for five categories of IPD: cases caused by all serotypes, cases caused by PCV7 serotypes (4, 6B, 9V, 14, 18C, 19F, and 23F), cases caused by three additional PCV10 serotypes (1, 5, and 7F), cases caused by three additional PCV13 serotypes (3, 6A, and 19A), and cases caused by non-PCV serotypes. To assess the impact of PCV, the study period was divided into the pre-vaccination period 2007–2008 and post-vaccination period 2009–2017, which was divided into three three-year parts: 2009–2011, 2012–2014, and 2015–2017. Analysis of differences between periods was based on the Poisson regression model where the population numbers were handled as an offset.

**Results:**

The annual incidence of IPD in children under 5 years of age caused by all serotypes has had a downward trend since 2007: it dropped from 8.52/100 000 in 2007 to 2.67/100 000 in 2017, with slight increases in 2010 and 2013. All three post-vaccination periods show significantly lower (p<0.001) incidences in comparison to the pre-vaccination period, but they do not statistically significantly differ from each other.

**Conclusions:**

IPD surveillance data in the Czech Republic show that after the introduction of PCV vaccination of infants, there has been a significant decrease in the IPD incidence of children under 5 years of age. Continued IPD surveillance is essential to monitor for possible post-vaccination serotype replacement.

## Introduction

*Streptococcus pneumoniae* remains the most widespread cause of vaccine preventable bacterial disease worldwide. WHO estimates that pneumococcal disease accounted for 476 thousand of 8.8 million deaths of children under 5 years of age in 2008 [1].

Pneumococcus can be present on the mucous membranes of the upper respiratory tract without causing infection or can cause disease, from milder (sinusitis, otitis) to severe (pneumonia, meningitis, sepsis). Severe forms of infection with the detection of *Streptococcus pneumoniae* from a normally sterile body site are considered as invasive pneumococcal disease (IPD) according to the European case definition [2].

The nationwide surveillance of IPD was implemented in the Czech Republic in 2007 and provides internationally comparable epidemiological data, which are reported to the European Surveillance System (TESSy) (https://tessy.ecdc.europa.eu/tessyweb). The overall incidence of IPD in the Czech Republic is analysed annually and published in the Bulletin of the Centre for Epidemiology and Microbiology (in Czech: Zprávy CEM) (http://www.szu.cz/publikace/zpravy-epidemiologie-a-mikrobiologie).

Almost 100 serotypes of *S*. *pneumoniae* have been described based on antigenic differences in the polysaccharide capsule. They are identified by conventional serological methods or polymerase chain reaction (PCR). Initially, a 23-valent pneumococcal polysaccharide vaccine (PPV) was developed from polysaccharide capsule antigens, but it only conferred short-term immunity and it could not be used in children under 2 years of age as it was not immunogenic in this age group. This disadvantage was removed by designing pneumococcal polysaccharide conjugate vaccines (PCV), starting with the 7-valent vaccine (PCV7), followed by the 10-valent vaccine (PCV10) and 13-valent vaccine (PCV13).

PCV7 was registered in the Czech Republic in 2005 and infants with underlying diseases were vaccinated. PCV10 and PCV13 were registered in 2009 and 2010, respectively. In 2009, PCV7 and PCV10 were available without indication, i.e. underlying disease, on the private market. In 2010, PCV10 and PCV13 were integrated into the national immunization program as a recommended vaccine for small children covered by the health insurance system, but only PCV10 is fully covered by insurance. In the case of PCV13 there is co-payment paid by parents. In the 3+1 immunization series (at the age of 2, 3, 4, and 12 months), PCV10 and PCV13 were applied equally, with no differences in individual regions of the country.

The aim of this study is to analyse the impact of PCV vaccination of infants on the incidence of IPD in children under 5 years of age in the Czech Republic.

## Material and methods

### Identification and serotyping of *S*. *pneumoniae*

*S*. *pneumoniae* isolates cultured from IPD cases were referred to the National Reference Laboratory for Streptococcal Infections (NRL) from the field laboratories. All *S*. *pneumoniae* IPD strains received were identified by the NRL based on their morphology, optochin susceptibility, bile solubility, and latex agglutination. Typing of all *S*. *pneumoniae* IPD strains received was performed using the Quellung reaction (SSI Diagnostice A/S, Denmark) in combination with end-point multiplex polymerase chain reaction (mPCR) followed by agarose electrophoresis [3, 4]. The genes used were capsular determinants typical for a serogroup or serotype of *S*. *pneumoniae*: *cps*A, *wzy*, *cps*H, *cps*I, *cap*B, *wci*Y, *cps*K, *cps*G, *gal*U, *wci*P, *cps*O, *wci*, N*beta*, *wcw*L, *wer*G, *wzx*, and *wci*L (The Centers for Disease Control and Prevention, Atlanta, USA, http://www.cdc.gov/ncidod/biotech/strep/pcr.htm).

DNA extraction from bacterial cultures was performed using the QIAmp DNA Mini Kit (Qiagen, FRG) according to the manufacturer’s protocol for DNA extraction from bacteria (http://www.qiagen.com/Products/Catalog/Sample-Technologies/DNA-Sample-Technologies/Genomic-DNA/QIAamp-DNA-Mini-Kit#technicalspecification).

### Surveillance of invasive pneumococcal disease

The nationwide IPD surveillance program was implemented in 2007 in cooperation between the NRL, regional epidemiologists, microbiologists, and infectious disease specialists. IPD isolates are referred to the NRL in accordance with Czech legislation. Isolates from over 90% of patients diagnosed with IPD of all age groups were referred to the NRL for confirmation and typing in the study period (http://www.szu.cz/publikace/zpravy-epidemiologie-a-mikrobiologie). IPD cases are reported by epidemiologists to the National Infectious Disease Reporting System. The surveillance database is created by merging data from the NRL database and the National Infectious Disease Reporting System, with the exclusion of duplicates. The NRL checks the data obtained and quarterly reminds the epidemiologists to report IPD cases to the National Infectious Disease Reporting System. The case definition of IPD is in agreement with the EU case definition [2]; it includes detection of *S*. *pneumoniae* by culture or PCR from a normally sterile body site as a laboratory criterion for diagnosis.

The surveillance covers the population of the whole Czech Republic, and the mid-year population numbers were derived from the Czech Statistical Office data (https://www.czso.cz/csu/czso/vekove-slozeni-obyvatelstva-2019). The numbers of children in the target group ranged between years from 500 to 600 thousand (S1 Table).

Authors had no access to identifying/personal details when accessing the records from National Infectious Disease Reporting System. All data were analysed anonymously.

The present study includes all IPD cases reported in children aged 0–4 years within the surveillance program in 2007–2017. The impact of PCV is analysed for five categories of IPD: cases caused by all serotypes, cases caused by PCV7 serotypes (4, 6B, 9V, 14, 18C, 19F, and 23F), cases caused by three additional PCV10 serotypes (1, 5, and 7F), cases caused by three additional PCV13 serotypes (3, 6A, and 19A), and cases caused by non-PCV serotypes.

To assess the impact of PCV, the study period was divided into the pre-vaccination period 2007–2008 and post-vaccination period 2009–2017. The latter nine-year period was divided into three three-year parts (2009–2011, 2012–2014, and 2015–2017) to evaluate changes in distribution of serotypes with decreasing vaccine coverage of target age group since 2010.

### Statistical analysis

IPD is characterized by counts of events and rates per 100 000 population of the target age group. Analysis of differences between periods was based on the Poisson regression model where the population numbers were handled as an offset [5]. The pre-vaccination incidence is used as a reference and is compared to those in the following periods. The results are presented in the form of incidence rate ratios (IRR) and corresponding 95% confidence intervals (95% CI). The significance level was set at 0.05 for all analyses. The Stata statistical software, release 14.2 (Stata Corp LP, College Station, TX, USA) was used for the calculation.

## Results

**Fig 1:**
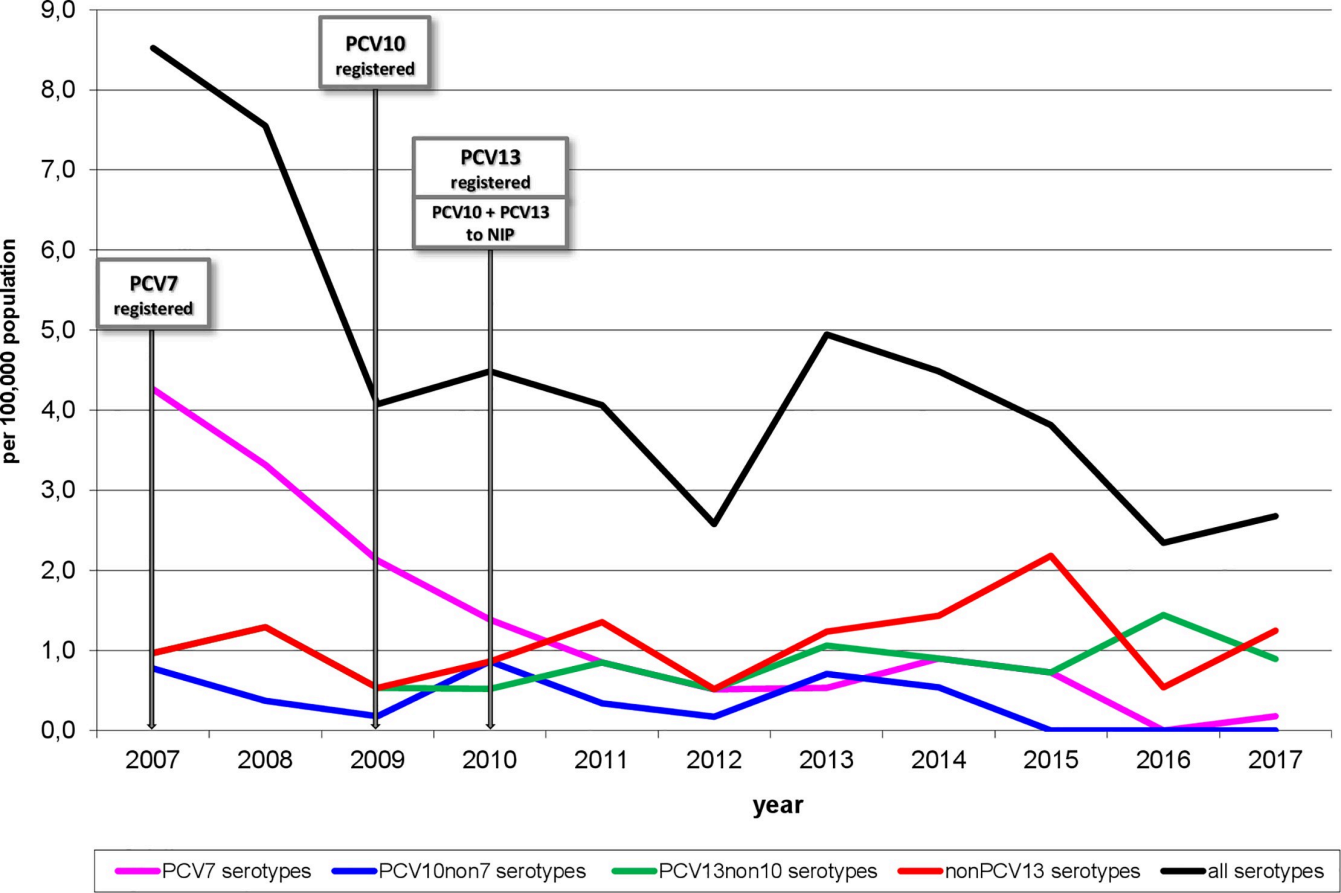
Incidence of invasive pneumococcal disease, under 5 years of age, according to serotypes, Czech Republic, 2007–2017, surveillance data.

The absolute numbers of all cases of IPD in children under 5 years of age ranged from 41 to 44 cases per year in the pre-vaccination period, decreasing to 15–28 cases per year in the post-vaccination period. The annual incidence of IPD in children under 5 years of age caused by all serotypes has had a downward trend since 2007: it dropped from 8.52/100 000 in 2007 to 2.67/100 000 in 2017, with slight increases in 2010 and 2013 (Fig 1).

All three post-vaccination periods show significantly lower (p<0.001) incidences of IPD caused by all serotypes in comparison to the pre-vaccination period (Table 1), but they do not differ significantly from each other.

**Table 1:** Incidence rate ratios for invasive pneumococcal disease, under 5 years of age, all serotypes, comparison of pre-vaccination period with three post-vaccination periods.

period	IRR	95% CI for IRR	p-value
2009–2011	0.52	(0.38,0.72)	<0.001
2012–2014	0.50	(0.36,0.68)	<0.001
2015–2017	0.37	(0.26,0.52)	<0.001

IRR-incidence rate ratio

Cases caused by PCV7 serotypes (4, 6B, 9V, 14, 18C, 19F, and 23F) dropped from 4.26 to 0.18/100 000 in the study period (Fig 1). All three post-vaccination periods showed significantly lower incidences (p<0.001) in comparison to the pre-vaccination period, with the incidence rate ratios being considerably inferior to 1 (Table 2). A significant decrease was also observed between the periods 2009–2011 and 2012–2014 (p = 0.026), but the difference between the periods 2012–2014 and 2015–2017 was not statistically significant (p = 0.156).

**Table 2:** Incidence rate ratios for invasive pneumococcal disease, under 5 years of age, PCV7 serotypes, comparison of pre-vaccination period with three post-vaccination periods.

period	IRR	95% CI for IRR	p-value
2009–2011	0.38	(0.23,0.63)	<0.001
2012–2014	0.17	(0.09,0.33)	<0.001
2015–2017	0.08	(0.03,0.20)	<0.001

IRR-incidence rate ratio

The annual incidence of IPD caused by three additional PCV10 serotypes (1, 5, and 7F) was low, ranging from 0.17 to 0.86/100 000, reaching zero in the last three years (Fig 1). Thus there was a significant difference in the annual incidence between the last post-vaccination period and the pre-vaccination period (p = 0.004) (Table 3).

**Table 3:** Incidence rate ratios for invasive pneumococcal disease, under 5 years of age, PCV10 non-PCV7 serotypes, comparison of pre-vaccination period with three post-vaccination periods.

period	IRR	95% CI for IRR	p-value
2009–2011	0.81	(0.28,2.35)	0.704
2012–2014	0.83	(0.29,2.39)	0.728
2015–2017	0.00	-	0.004

IRR-incidence rate ratio

Similar low annual incidences were found for the other three additional PCV13 serotypes (3, 6A, and 19A), ranging from 0.52 to 1.44/100 000 (Fig 1). After a drop in the annual incidence in the period 2009–2011 as compared to the pre-vaccination period, a slight upward trend is seen, but the difference does not reach statistical significance (Table 4). There is no significant difference in incidence between any two of the four study periods.

**Table 4:** Incidence rate ratios for invasive pneumococcal disease, under 5 years of age, PCV13 non-PCV10 serotypes, comparison of pre-vaccination period with three post-vaccination periods.

period	IRR	95% CI for IRR	p-value
2009–2011	0.56	(0.25,1.27)	0.165
2012–2014	0.72	(0.34,1.57)	0.414
2015–2017	0.90	(0.43,1.89)	0.782

IRR-incidence rate ratio

The annual incidence of IPD cases caused by non-PCV13 serotypes ranged from 0.52 to 2.18/100 000 and has a slight upward trend in the post-vaccination period (Fig 1). However, there is no significant difference between any two of the study periods (Table 5).

**Table 5:** Incidence rate ratios for invasive pneumococcal disease, under 5 years of age, non-PCV13 serotypes, comparison of pre-vaccination period with three post-vaccination periods.

period	IRR	95% CI for IRR	p-value
2009–2011	0.81	(0.39,1.72)	0.591
2012–2014	0.93	(0.45,1.93)	0.850
2015–2017	1.17	(0.58,2.36)	0.669

IRR-incidence rate ratio

The total number of all IPD cases in children under 5 years of age was 275. In 225 (81.8%) of them the serotype was identified (S2 Table). From those 225 IPD cases, 81 (36%) were caused by PCV7 serotypes, 22 (9.8%) by additonal PCV10 serotypes, 54 (24%) by additional PCV13 serotypes, and 68 (30.2%) by non-PCV13 serotypes. The most frequent serotypes in children under 5 years of age in the whole period were serotype 14 and serotype 3 (both causing 24 cases), but showing different behavior: serotype 14 decreased in post-vaccination periods, while serotype 3 showed a stable trend with a slight increase in the last post-vaccination period (S2 Table). The second most frequent was serotype 19A (causing 19 cases) which showed an increasing trend in the post-vaccination period. The next most frequent was serotype 1 (causing 15 cases) showing a stable trend in two post-vaccination periods and reaching zero in the last one. The remaining serotypes in descending order were as follows: serotypes 6B, 23F, 6A, 9V, 19F, 18C, 7F, and 4 (covered by PCVs), and serotypes 15C, 15B, 17F, and 25A (not covered by PCVs). The rest of the non-PCVs serotypes were found in number lower than 5 for the whole period followed (S2 Table).

The number of IPD cases in children 0–11 months of age with identified serotype was 62 (S3 Table), with the most frequent serotype 19F (showing a decreasing trend in the last postvaccination period). In second place were serotypes 3, 18C, 19A. The number of IPD cases in children of 1 year of age was 59 (S4 Table), with the most frequent serotype 14 (showing a decreasing trend in the post-vaccination periods). Serotype 6B was the next most frequent, followed by serotypes 3 and 6A. The number of IPD cases in children of 2 years of age was 22 (S5 Table), with serotype 19A seen most frequently. The number of IPD cases in children of 3 years of age was 42 (S6 Table), with the most frequent serotype 3, followed by serotype 23F, and then serotype 14. The number of IPD cases in children of 4 years of age was 40 (S7 Table). with the most frequent serotype 1, followed by serotype 14, and then serotype 3.

**Fig 2:**
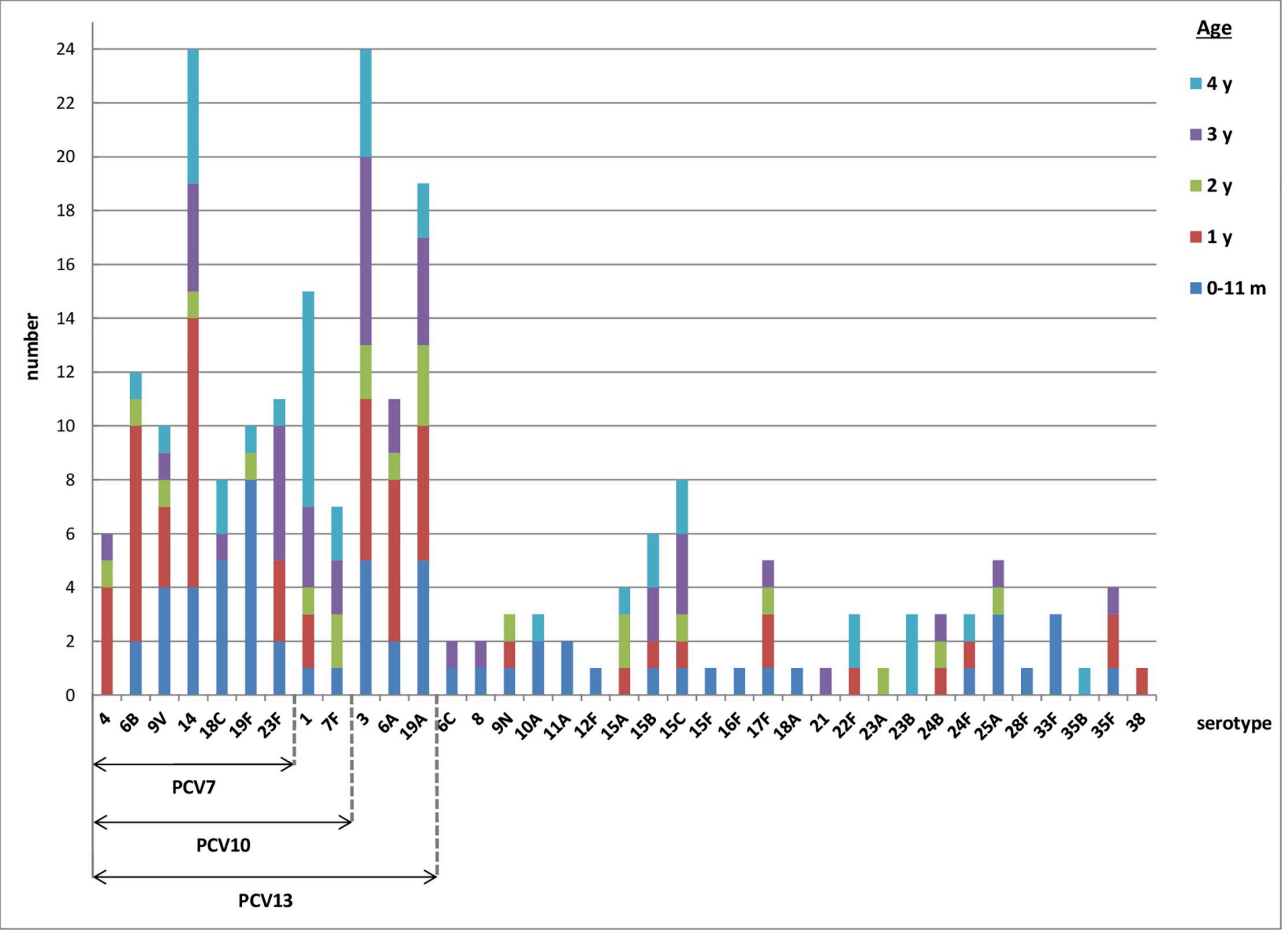
Distribution of serotypes causing invasive pneumococcal disease, under 5 years of age, Czech Republic, 2007–2017, surveillance data.

The PCV7 serotypes predominated in children from 0–11 months and 1 year olds, the additional PCV10 serotypes were the most frequent in 4 years olds and the additional PCV13 serotypes were distributed in all ages under 5 years old (Fig 2).

## Discussion

The results of our study show decreased incidence of IPD in children under 5 years of age caused by all serotypes, consistent with the data reported by others [6–9]. A high effectiveness of PCV7 and PCV13 in the prevention of IPD caused by vaccine serotypes was demonstrated in England, with the exception of serotype 3, which was responsible for progressive increase in the post-vaccination incidence of IPD, and serotype 19A persisting in the post-vaccination period [10].

The initial increase in the incidence of IPD caused by non-PCV13 serotypes in the Czech Republic suggests serotype replacement and is in line with the data reported from other European countries [11–13]. In contrast, a study of the impact of PCV13 on IPD in children under 15 years of age in Madrid did not show serotype replacement in the post-vaccination period [14].

Post-vaccination serotype replacement was also shown in a large international study with the participation of the Czech Republic. That study compared IPD surveillance data from 21 sites obtained ≥2 years before and ≥1 year after the introduction of PCV7. A significant drop in the overall incidence of IPD caused by PCV7 serotypes was observed in small children. Increases in non-PCV types IPD occurred in most sites, with variable magnitudes [15].

The Czech Republic participates in the European project SpIDnet, the first results of which showed that in the participating countries (Czech Republic, France, Ireland, Norway, Sweden, and Spain), four years after the implementation of PCV13 alone or PCV10 and PCV13, the incidence rate ratios (IRR) of IPD in children under 5 years of age were 0.53 for cases caused by all serotypes (95% CI 0.43–0.65), 0.16 for cases caused by PCV7 serotypes (0.07–0.040), 0.41 for cases caused by three additional PCV10 serotypes (0.07–0.042), 0.41 for cases caused by three additional PCV13 serotypes (0.25–0.69), and 1.62 for cases caused by non-PCV13 serotypes (1.09–2.42). From these data, it is evident that the overall incidence of IPD decreased due to the drop in cases caused by vaccine serotypes. On the other hand, the incidence of IPD caused by non-vaccine serotypes is on the rise, which suggests serotype replacement [16].

The serotypes causing IPD indicated in replacement are diverse among countries implementing PCVs, with a heterogeneity in magnitude of replacement and serotypes involved [17]. The main non-PCV13 serotypes were 12F, 24F, and 12B in the United Kingdom in 2015 [18] and 10A, 12F, 23B, 24F, and 38 in Germany in the vaccination period [19]. These non-PCV13 serotypes were rare in our study (S2 Table): 10A (3 IPD cases), 12B (no IPD case), 12F (one IPD case), 23B (3 IPD cases), 24F (3 IPD cases).

A meta-analysis of the serotype distribution in the post-PCV era (in 14 countries where PCV7 was administered and 24 countries where PCV10 or PCV13 have been introduced) showed that serotype 19A was the predominant cause of childhood IPD, accounting for 21.8% of cases [20], which correlates with our findings: serotype 19A was in second position in our study (S2 Table). Non-PCV13 serotypes (predominantly 22F, 12F, 33F, 24F, 15C, 15B, 23B, 10A and 38) contributed to 42.2% of IPD in the total meta-analysis results, with regional differences: 71.9% in Europe, 57.8% in North America, 45.9% in Western Pacific and 28.5% in Latin America. In our study, 30.2% of IPD cases were caused by non-PCV13 serotypes, predominantly 15C, 15B, 17F and 25A (S2 Table).

Our study has considerable advantages, namely the high proportion of successfully serotyped causative isolates and a highly sensitive national IPD surveillance program, which provides valid and internationally comparable data. Consistent methodology of IPD surveillance applied in the Czech Republic since 2007 allows vaccine impact assessment.

The sensitivity of IPD surveillance in the Czech Republic was analysed by the capture-recapture method for the period 2008–2013 [21]. The overall system sensitivity increased from 81% to 99%, the laboratory sensitivity rose from 72% to 87%, and the reporting sensitivity jumped from 31% to 89%. A major role in increased IPD surveillance sensitivity was played by the quarterly reminder for epidemiologists to report IPD cases implemented since 2011. Due to increased susceptibility of IPD surveillance, the vaccine impact on the incidence of IPD may be underestimated.

After initial high vaccine coverage of the target age group by PCV10 and PCV13 applied equally in the 3+1 immunization series, a downward trend was seen: 78% in 2010, 81.0% in 2011, 80.0% in 2012, 75.0% in 2013, 73.9% in 2014, 70.6% in 2015, 67.9% in 2016, and 67.5% in 2017. The reasons for the decrease of vaccine uptake could be the increased influence of the anti-vaccine lobby in the country and the fact that PCVs were only recommended, rather than mandatory, in the NIP. It can be assumed that maintaining a high vaccination coverage of the target age group would have resulted in greater reduction in the incidence of IPD in children under 5 years of age.

The use of PCV10 versus PCV13 is under discussion in the Czech Republic. The switch from the use of PCV10 and PCV13 equally, to use of PCV13 only, could cause the decrease of persisting serotypes covered by this vaccine. However, the persistence of serotypes 3 and 19A is reported from countries implementing the PCV13 vaccination [22–25].

## Conclusions

IPD surveillance data in the Czech Republic show that after the introduction of PCV vaccination of infants, there has been a significant decrease in the IPD incidence of children under 5 years of age. Continued IPD surveillance is essential to monitor for possible post-vaccination serotype replacement.

## Supporting information

S1 Table Mid-year population and birth cohort, Czech Republic, 2007–2017. (PDF)

S2 Table Distribution of serotypes causing IPD in children under 5 years of age, Czech Republic, 2007–2017. (PDF)

S3 Table Distribution of serotypes causing IPD in children 0–11 months of age, Czech Republic, 2007–2017. (PDF)

S4 Table Distribution of serotypes causing IPD in children 1 year of age, Czech Republic, 2007–2017. (PDF)

S5 Table Distribution of serotypes causing IPD in children 2 years of age, Czech Republic, 2007–2017. (PDF)

S6 Table Distribution of serotypes causing IPD in children 3 years of age, Czech Republic, 2007–2017. (PDF)

S7 Table Distribution of serotypes causing IPD in children 4 years of age, Czech Republic, 2007–2017. (PDF)
