# Supplementary material for: Impact of pneumococcal conjugate vaccine on invasive pneumococcal disease in children under 5 years of age in the Czech Republic
Source: PLoS One. 2021 Feb 26;16(2):e0247862. doi: 10.1371/journal.pone.0247862 (PMC7909631; doi:10.1371/journal.pone.0247862)
Supplement: S1 Table — (PDF) [file pone.0247862.s001.pdf]

S1 Table

Mid-year population and birth cohort, Czech Republic, 2007-2017.

**Year**	**Age group**		
	**Total**	**0-4 years**	**Birth cohort**
2007	10322689	504394	110023
2008	10429692	530098	117264
2009	10491492	554085	119304
2010	10517247	571974	118067
2011	10496672	590354	113970
2012	10509286	586159	108724
2013	10510719	573939	107763
2014	10524783	561656	108384
2015	10542942	553617	110356
2016	10565284	552453	111660
2017	10589526	557800	113379

Source:
Czech Statistical Office
https://www.czso.cz/csu/czso/age-distribution-of-the-population-2019
Accessed 16-12-2020
