# Supplementary material for: Impact of pneumococcal conjugate vaccine on invasive pneumococcal disease in children under 5 years of age in the Czech Republic
Source: PLoS One. 2021 Feb 26;16(2):e0247862. doi: 10.1371/journal.pone.0247862 (PMC7909631; doi:10.1371/journal.pone.0247862)
Supplement: S2 Table — (PDF) [file pone.0247862.s002.pdf]

S2 Table

Distribution of serotypes causing IPD in children under 5 years of age

Surveillance data, Czech Republic, 2007 - 2017.

Serotype	2007	2008	2009	2010	2011	2012	2013	2014	2015	2016	2017	Total
**1**	4	1	0	3	1	1	3	2	0	0	0	**15**
**3**	2	4	1	2	2	0	1	3	2	6	1	**24**
**4**	2	2	0	1	0	0	0	1	0	0	0	**6**
**6A**	2	2	2	1	1	1	0	0	0	1	1	**11**
**6B**	3	1	3	3	0	0	0	1	1	0	0	**12**
**6C**	0	0	0	0	0	0	0	1	1	0	0	**2**
**7F**	0	1	1	2	1	0	1	1	0	0	0	**7**
**8**	0	1	0	0	0	0	0	1	0	0	0	**2**
**9N**	1	1	0	0	0	0	0	0	1	0	0	**3**
**9V**	1	2	2	1	0	0	1	2	1	0	0	**10**
**10A**	1	0	0	1	0	0	0	0	1	0	0	**3**
**11A**	0	0	0	0	0	0	1	0	0	0	1	**2**
**12F**	0	0	0	1	0	0	0	0	0	0	0	**1**
**14**	9	4	3	2	2	1	1	0	1	0	1	**24**
**15A**	0	0	0	0	1	0	0	1	2	0	0	**4**
**15B**	0	1	0	1	1	0	2	0	0	0	1	**6**
**15C**	1	1	1	1	1	1	0	0	1	0	1	**8**
**15F**	0	0	0	0	0	0	1	0	0	0	0	**1**
**16F**	0	0	0	0	0	0	0	1	0	0	0	**1**
**17F**	1	1	0	0	1	0	1	1	0	0	0	**5**
**18A**	0	0	1	0	0	0	0	0	0	0	0	**1**
**18C**	2	2	0	1	2	0	0	0	1	0	0	**8**
**19A**	1	1	0	0	2	2	5	2	2	1	3	**19**
**19F**	2	4	1	0	0	1	1	1	0	0	0	**10**
**21**	0	0	0	0	0	0	0	0	1	0	0	**1**
**22F**	0	0	1	0	0	0	0	1	1	0	0	**3**
**23A**	0	0	0	0	0	1	0	0	0	0	0	**1**
**23B**	0	0	0	0	0	0	0	1	2	0	0	**3**
**23F**	3	3	3	0	1	1	0	0	0	0	0	**11**
**24B**	0	0	0	0	0	0	0	0	1	2	0	**3**
**24F**	0	0	0	0	1	1	1	0	0	0	0	**3**
**25A**	1	0	0	0	3	0	0	0	0	0	1	**5**
**28F**	0	0	0	0	0	0	0	0	0	0	1	**1**
**33F**	0	1	0	1	0	0	0	0	1	0	0	**3**
**35B**	0	0	0	0	0	0	0	0	0	0	1	**1**
**35F**	0	1	0	0	0	0	0	1	0	1	1	**4**
**38**	0	0	0	0	0	0	1	0	0	0	0	**1**
**IPD typed**	**36**	**34**	**19**	**21**	**20**	**10**	**20**	**21**	**20**	**11**	**13**	**225**
**IPD not typed**	8	7	4	5	4	5	8	4	1	2	2	50
**IPD total**	**44**	**41**	**23**	**26**	**24**	**15**	**28**	**25**	**21**	**13**	**15**	**275**

blue = PCV7 serotypes

green = additional PCV10 serotypes

yellow = additional PCV13 serotypes
