# Supplementary material for: Impact of pneumococcal conjugate vaccine on invasive pneumococcal disease in children under 5 years of age in the Czech Republic
Source: PLoS One. 2021 Feb 26;16(2):e0247862. doi: 10.1371/journal.pone.0247862 (PMC7909631; doi:10.1371/journal.pone.0247862)
Supplement: S4 Table — (PDF) [file pone.0247862.s004.pdf]

S4 Table

Distribution of serotypes causing IPD in children 1 year of age

Surveillance data, Czech Republic, 2007 - 2017.

Serotype	2007	2008	2009	2010	2011	2012	2013	2014	2015	2016	2017	Total
**1**	1	1	0	0	0	0	0	0	0	0	0	**2**
**3**	0	2	0	0	0	0	1	2	0	1	0	**6**
**4**	2	1	0	0	0	0	0	1	0	0	0	**4**
**6A**	1	0	2	0	1	0	0	0	0	1	1	**6**
**6B**	2	0	2	3	0	0	0	0	1	0	0	**8**
**9N**	0	1	0	0	0	0	0	0	0	0	0	**1**
**9V**	0	0	1	1	0	0	0	1	0	0	0	**3**
**14**	4	2	2	1	0	0	0	0	1	0	0	**10**
**15A**	0	0	0	0	1	0	0	0	0	0	0	**1**
**15B**	0	0	0	1	0	0	0	0	0	0	0	**1**
**15C**	0	0	0	0	0	0	0	0	1	0	0	**1**
**17F**	1	0	0	0	0	0	0	1	0	0	0	**2**
**19A**	1	0	0	0	0	0	1	0	2	0	1	**5**
**22F**	0	0	1	0	0	0	0	0	0	0	0	**1**
**23F**	2	0	1	0	0	0	0	0	0	0	0	**3**
**24B**	0	0	0	0	0	0	0	0	0	1	0	**1**
**24F**	0	0	0	0	0	0	1	0	0	0	0	**1**
**35F**	0	0	0	0	0	0	0	1	0	0	1	**2**
**38**	0	0	0	0	0	0	1	0	0	0	0	**1**
**IPD typed**	**14**	**7**	**9**	**6**	**2**	**0**	**4**	**6**	**5**	**3**	**3**	**59**
**IPD not typed**	1	3	2	0	0	2	1	0	0	1	0	10
**IPD total**	**15**	**10**	**11**	**6**	**2**	**2**	**5**	**6**	**5**	**4**	**3**	**69**

blue = PCV7 serotypes

green = additional PCV10 serotypes

yellow = additional PCV13 serotypes
