# Supplementary material for: Impact of pneumococcal conjugate vaccine on invasive pneumococcal disease in children under 5 years of age in the Czech Republic
Source: PLoS One. 2021 Feb 26;16(2):e0247862. doi: 10.1371/journal.pone.0247862 (PMC7909631; doi:10.1371/journal.pone.0247862)
Supplement: S5 Table — (PDF) [file pone.0247862.s005.pdf]

S5 Table

Distribution of serotypes causing IPD in children 2 years of age

Surveillance data, Czech Republic, 2007 - 2017.

Serotype	2007	2008	2009	2010	2011	2012	2013	2014	2015	2016	2017	Total
**1**	0	0	0	0	0	0	1	0	0	0	0	**1**
**3**	0	0	0	0	0	0	0	0	0	2	0	**2**
**4**	0	1	0	0	0	0	0	0	0	0	0	**1**
**6A**	0	0	0	1	0	0	0	0	0	0	0	**1**
**6B**	0	0	1	0	0	0	0	0	0	0	0	**1**
**7F**	0	0	0	2	0	0	0	0	0	0	0	**2**
**9N**	0	0	0	0	0	0	0	0	1	0	0	**1**
**9V**	0	1	0	0	0	0	0	0	0	0	0	**1**
**14**	0	0	0	0	1	0	0	0	0	0	0	**1**
**15A**	0	0	0	0	0	0	0	0	2	0	0	**2**
**15C**	0	0	0	0	0	1	0	0	0	0	0	**1**
**17F**	0	1	0	0	0	0	0	0	0	0	0	**1**
**19A**	0	0	0	0	0	0	0	1	0	1	1	**3**
**19F**	1	0	0	0	0	0	0	0	0	0	0	**1**
**23A**	0	0	0	0	0	1	0	0	0	0	0	**1**
**24B**	0	0	0	0	0	0	0	0	0	1	0	**1**
**25A**	1	0	0	0	0	0	0	0	0	0	0	**1**
**IPD typed**	**2**	**3**	**1**	**3**	**1**	**2**	**1**	**1**	**3**	**4**	**1**	**22**
**IPD not typed**	0	0	0	0	0	2	2	0	1	1	0	6
**IPD total**	**2**	**3**	**1**	**3**	**1**	**4**	**3**	**1**	**4**	**5**	**1**	**28**

blue = PCV7 serotypes

green = additional PCV10 serotypes

yellow = additional PCV13 serotypes
