# Supplementary material for: Impact of pneumococcal conjugate vaccine on invasive pneumococcal disease in children under 5 years of age in the Czech Republic
Source: PLoS One. 2021 Feb 26;16(2):e0247862. doi: 10.1371/journal.pone.0247862 (PMC7909631; doi:10.1371/journal.pone.0247862)
Supplement: S7 Table — (PDF) [file pone.0247862.s007.pdf]

S7 Table

Distribution of serotypes causing IPD in children 4 years of age

Surveillance data, Czech Republic, 2007 - 2017.

Serotype	2007	2008	2009	2010	2011	2012	2013	2014	2015	2016	2017	Total
**1**	1	0	0	3	1	1	1	1	0	0	0	**8**
**3**	1	0	0	0	1	0	0	0	1	0	1	**4**
**6B**	1	0	0	0	0	0	0	0	0	0	0	**1**
**7F**	0	0	0	0	1	0	0	1	0	0	0	**2**
**9V**	1	0	0	0	0	0	0	0	0	0	0	**1**
**10A**	1	0	0	0	0	0	0	0	0	0	0	**1**
**14**	3	0	1	0	0	0	1	0	0	0	0	**5**
**15A**	0	0	0	0	0	0	0	1	0	0	0	**1**
**15B**	0	1	0	0	0	0	0	0	0	0	1	**2**
**15C**	0	0	1	0	1	0	0	0	0	0	0	**2**
**18C**	0	0	0	0	2	0	0	0	0	0	0	**2**
**19A**	0	0	0	0	1	1	0	0	0	0	0	**2**
**19F**	0	1	0	0	0	0	0	0	0	0	0	**1**
**22F**	0	0	0	0	0	0	0	1	1	0	0	**2**
**23B**	0	0	0	0	0	0	0	1	2	0	0	**3**
**23F**	0	1	0	0	0	0	0	0	0	0	0	**1**
**24F**	0	0	0	0	0	1	0	0	0	0	0	**1**
**35B**	0	0	0	0	0	0	0	0	0	0	1	**1**
**IPD typed**	**8**	**3**	**2**	**3**	**7**	**3**	**2**	**5**	**4**	**0**	**3**	**40**
**IPD not typed**	1	1	2	1	2	1	0	1	0	0	0	9
**IPD total**	**9**	**4**	**4**	**4**	**9**	**4**	**2**	**6**	**4**	**0**	**3**	**49**

blue = PCV7 serotypes
green = additional PCV10 serotypes
yellow = additional PCV13 serotypes
